# A Case Study on the Efficacy of Hypo-Osmotic Swelling Test and Assisted Hatching in Overcoming Necrozoospermia Challenges

**DOI:** 10.7759/cureus.54912

**Published:** 2024-02-26

**Authors:** Shivani Khemani, Akash More, Jarul Shrivastava, Namrata Choudhary

**Affiliations:** 1 Clinical Embryology, Datta Meghe Institute of Higher Education and Research, Wardha, IND

**Keywords:** gnrh short antagonist protocol, gnrha trigger, mechanical assisted hatching, necrozoospermia, infertility, hypo-osmotic test, assisted hatching, pcos

## Abstract

This case report examines the difficulties faced by a couple with 11 years of primary infertility. Based on the diagnostic evaluation, it was determined that the male was a necrozoospermia patient, while the female had unilateral cornual blockage and polycystic ovarian syndrome (PCOS) with diabetes mellitus (DM) symptoms identified. A comprehensive approach was used in the treatment for the female patient, which included a gonadotropin-releasing hormone (GnRH) short antagonist protocol, a GnRH agonist (GnRHa) trigger, assisted hatching (AH), and the use of the hypo-osmotic swelling test (HOST) to gauge the viability of the sperm. The successful outcome, as evidenced by the increasing levels of beta-human chorionic gonadotropin (β-hCG) and a successful embryo transfer, highlights the effectiveness of a customized and multifaceted approach in managing intricate infertility problems. This instance offers insightful information about the way modern reproductive technologies can be successfully integrated with specialized treatment plans to achieve successful outcomes in difficult cases of infertility.

## Introduction

About 85% of infertility cases have identified causes. Male-factor infertility, ovulatory dysfunction, and tubal disease are the three most prevalent causes of infertility. About 25% of cases of infertility are caused by ovarian disorders; polycystic ovarian syndrome affects 70% of anovulating women [1]. Five to ten percent of women in the fertile-age group are affected by polycystic ovary syndrome (PCOS), which is characterized by a range of metabolic abnormalities (for instance, hyperandrogenism, obesity, insulin resistance), polycystic ovaries, and ovulatory dysfunction [2]. Based on clinical and biochemical evaluations of hyperandrogenism in conjunction with ultrasonography evaluations for polycystic-appearing ovaries and oligomenorrhea, PCOS is diagnosed. This is according to the Rotterdam criteria. However, this diagnosis may be accompanied by clinical features such as infertility, psychological conditions like anxiety and depression, obesity, and other aspects of the metabolic syndrome [3]. A growing percentage of PCOS-related infertile patients decide to try assisted reproductive technology (ART) for conception [4].

The ART field has advanced significantly over the past 40 years, with several different approaches undergoing significant changes. Even though ART's success rates are rising, some embryos created with it occasionally fail to implant successfully. Before implantation into the uterus, the embryo must break free from the zona pellucida (ZP), an outer glycoprotein coat, to result in a successful pregnancy. The term "embryo hatching" refers to this physiological process. One of the elements contributing to implantation failure may be the blastocyst's or ZP's inability to hatch as a result of abnormalities [5]. To address this issue, the process of assisted hatching (AH), which involves creating a hole in the embryo's outer layer and assisting its departure, is used in ART. Several AH methods, such as laser, mechanical, and chemical ones, have been used [6].

Assessing human sperm viability is one of the major components of semen analysis. A relatively simple sperm vitality test called the hypo-osmotic swelling test (HOST) is used to evaluate how well the spermatozoa's tail membrane functions. The sperm tails in a hypo-osmotic solution might or might not swell during the incubation of semen samples in the solution. The WHO's Fifth Manual states that these conditions are classified as necrozoospermia if the percentage of live cells is less than 58% [7,8].

## Case presentation

Patient information

This case report centers around a couple with 11 years of primary infertility who visited a fertility clinic in Nagpur division, Maharashtra, India. The patient's spouse was 45 years old, and the patient herself was 42 years old. The female was a housewife, and her husband was a hotel owner. The patient was suffering from severe weight gain, dysmenorrhea, and an irregular menstrual cycle. Neither partner had undergone any medical or surgical procedures in the past. They have been trying to conceive for the past 11 years. The female has been a patient with PCOS for the last eight years. There was a history of diabetes mellitus (DM) in the patient's family.

Physical examination

The female partner's body mass index (BMI) was 28 kg/m2, while the male partner's BMI was 24 kg/m2. The female patient's face showed abnormal hair growth, particularly in the area around her upper lips.

Investigation

Biochemistry and Hormonal Report

The female partner's report is shown below in Tables 1, 2.

**Table 1:** Biochemistry report of the female partner

Investigation	Observed value	Biological reference range
HbA1C	10.0	6.0 - 7.0% AIC NGSP
RBS-glucose-plasma random	194	70 - 150 mg%
Liver function test (LFT)		
Serum glutamic-pyruvate transaminase (SGPT)	38	<35 U/L
Serum glutamic-oxaloacetic transaminase (SGOT)	22	14 - 36 U/L
Alkaline phosphatase	116	20 - 140 U/L
Total protein	7.6	6.0 - 8.3 g/dL
Albumin	4.3	3.4 - 5.4 g/dL
Globulin	6.3	2.3 - 3.5 g/dL
Total bilirubin	0.6	0.2 - 1.3 mg/dL
Bilirubin conjugated (BC)	0.2	0.1 - 0.3 mg/dL
Bilirubin unconjugated (BU)	0.1	0.2 - 1.0 mg/dL
Urea	5	7.2 - 20 mg/dL
Creatinine	0.5	0.55 - 1.2 mg/dL
Sodium (Na+)	134	137 - 145 mmol/L
Potassium (K+)	4.7	3.2 - 5.3 mmol/L

**Table 2:** Hormonal profile of the female partner

Investigation	Observed	Reference range
Follicular stimulating hormone mIU/mL	3.13	3.5 - 12.5 mIU/mL
Anti-mullerian hormone (AMH) ng/mL	6.54	1.0 - 4.0 ng/mL
Prolactin ng/mL	18.2	2 - 29 ng/mL
Estradiol pg/mL	33.76	30 - 400 pg/mL
Luteinizing hormone (LH) mIU/mL	15.8	2 - 15 mIU/mL
Testosterone ng/mL	1.05	0.15 - 0.7 ng/mL
Estradiol pg/mL	33.76	30 - 400 pg/mL

All reports were within the normal range for the male partner.

Ultra-Sound Findings

**Figure 1:**
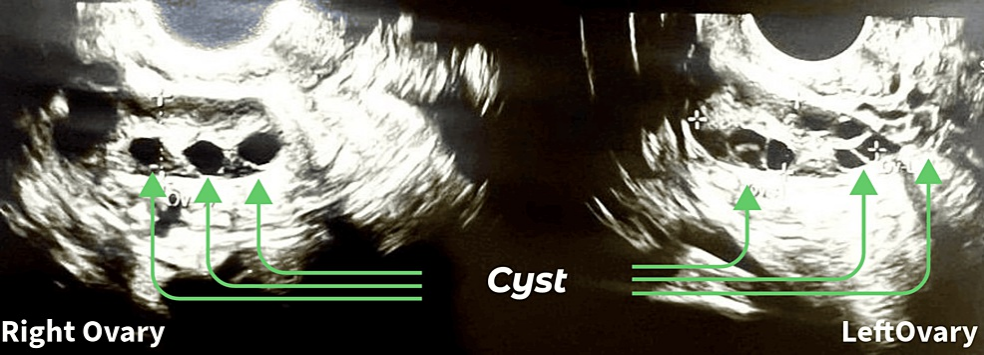
USG of female partner showing multiple cysts USG: ultrasound sonography

The right fallopian tube was found to be patent during the sono-hysterosalpingography, while the left fallopian tube had a unilateral cornual blockage. There were several cysts visible in both ovaries, as shown in Figure 1.

Semen Analysis

Semen analysis was carried out every two months with abstinence of three to four days. The average of those three evaluations revealed that the volume was 1.6 ml, the count was 30 million/ml, the motility was 20%, the normal morphology was 4%, and the total vital sperm was 30%. These were carried out according to the WHO guidelines.

Diagnosis

The husband's necrozoospermia, along with other factors in the female partner such as unilateral cornual blockage and PCOS, contribute to the couple's challenges with infertility. Furthermore, biochemical reports point to DM as a possible cause.

Treatment

**Figure 2:**
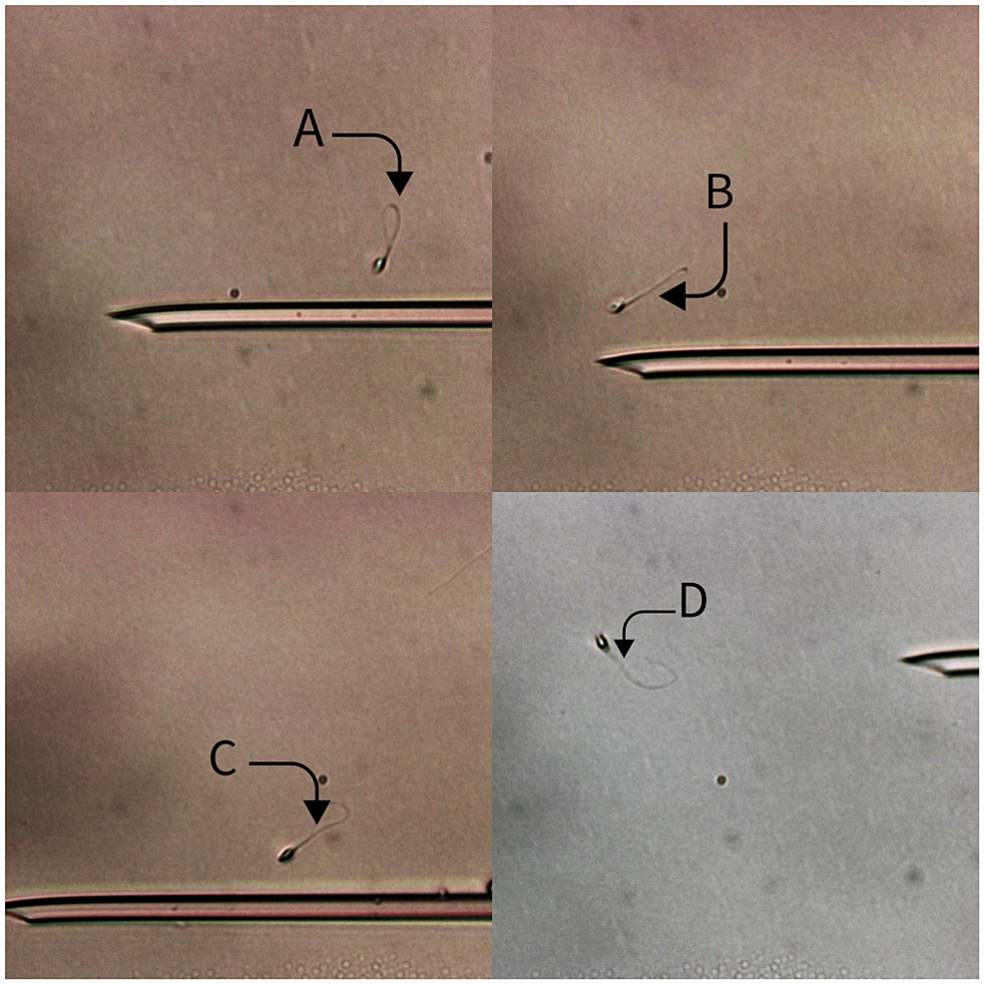
Sperm were selected using the hypo-osmotic swelling test (HOST). Arrow A-D pointed towards sperm with coiled tails, indicating an intact cell membrane The original picture was captured by the author of this article

**Figure 3:**
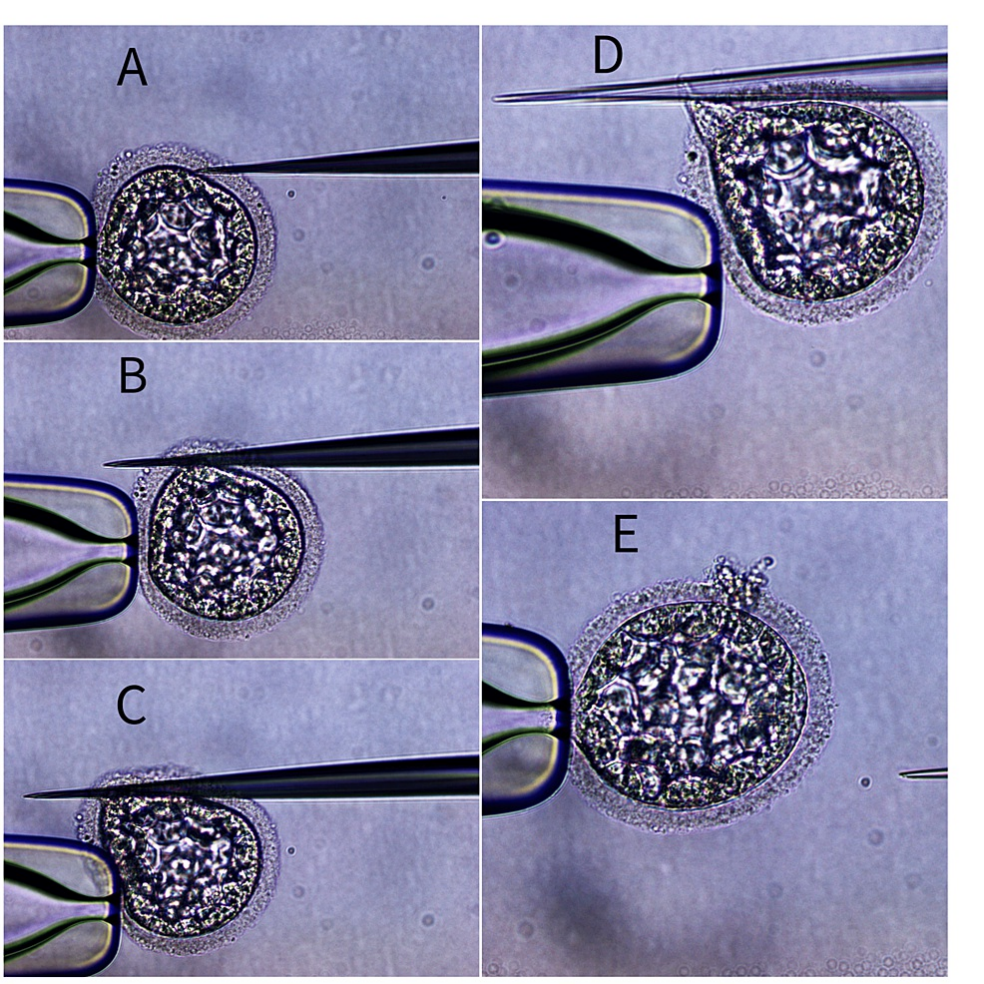
Image showing step-wise (A-E) mechanical-assisted hatching (MAH) of the blastocyst The original picture was captured by the author of this article

The gonadotropin-releasing hormone (GnRH) short antagonist protocol along with 250 mg of metformin capsule was initiated ten days prior to the ovum pick-up (OPU) following counseling and the acquisition of written informed consent from the patients. This protocol is commonly used for PCOS patients. Thirty-six hours before OPU, a GnRH agonist (GnRHa) was administered as a trigger. After retrieving nine oocytes from the right ovary, denudation revealed that two were in metaphase I (MI), six were in metaphase II (MII), and one was in the germinal vesicle (GV) stage. Concurrently, because the patient was a male with necrozoospermia, HOST was performed. This involved processing the semen and keeping the sperm in a hypoosmotic solution for less than five minutes. The sperms were then selected and rinsed in sperm preparation medium as soon as the tail began to swell and curl (Figure 2). Of the six MII oocytes, five blastocysts formed on day five and were frozen. A month later, the transfer of the embryos was planned; two of the five frozen embryos were thawed, and a few hours after the thawing, it was noticed that the ZP was thick, but due to the unavailability of the hatching preparations, the embryo was transferred. The results of the beta-human chorionic gonadotropin (β-hCG) test were negative after 14 days. The thickening of ZP may have contributed to the patient's infertility. We therefore decided to perform mechanical assisted hatching (MAH) after discussing the option with the patient and receiving written informed consent. Approximately a month later, the embryo transfer was planned; this time, two hours after the embryos had been thawed, MAH was carried out (Figure 3) on both embryos and after two hours of MAH, the embryos were transferred.

Follow-up

The patient was instructed to continue taking metformin, take adequate rest, and not engage in any physically exhausting activity or heavy lifting following embryo transfer. When serum β-hCG was tested after 14 days, the result showed 320 mIU/ml, which was positive. The patient was then instructed to take progesterone, iron supplements, metformin, and multivitamin medications. Additionally, routine check-ups every two weeks were asked of her.

## Discussion

Numerous studies have demonstrated that ovarian hyperstimulation syndrome (OHSS) is highly likely to occur in PCOS patients. For patients with PCOS, a GnRH antagonist protocol often serves as the first option for controlled ovarian stimulation (COS) to lower the risk of OHSS [4,9]. We also used a GnRH antagonist protocol to stimulate our patient, who also had PCOS, and this proved beneficial for the patient. Regarding the final oocyte maturation, we used GnRHa as an alternative trigger, which has also been demonstrated to be beneficial for PCOS patients, in place of hCG, which extends the luteotropic action of the hormone hCG and raises the chances of OHSS in PCOS patients [10].

We employed HOST as some articles recommend using it to select sperm from patients with necrozoospermia because it is a test that only detects swelling in living sperm that have intact membranes when exposed to a hypo-osmotic environment. Sperm tail curvature reflects the inflow of fluid into cells that are viable. This test's ability to preserve spermatozoa, which permits their use in ART, is one of its benefits [8,11].

Among several AH techniques, our patient underwent MAH owing to her advanced age. Additionally, due to the thick ZP as seen in the embryos, it's possible that this contributed to her infertility, as some articles suggest using AH for the successful implantation of embryos in patients with such characteristics [6]. The purpose of assisted hatching is to facilitate the implantation of the embryo into the uterine lining, which can potentially improve the chances of a successful pregnancy. The exact mechanism by which the AH procedure works is still unknown; however, the current hypothesis suggests that it weakens the ZP and encourages the embryo to escape and attach itself to the endometrial lining. The AH procedure is usually carried out on embryos that are of good quality, as it is believed that embryos of poor quality will not benefit from it. It is also used in cases where the ZP is thickened or the embryo has not been successfully implanted after previous attempts at embryo transfer [12,13].

## Conclusions

This case report concludes by demonstrating the effective handling of a primary infertility case that spanned 11 years. A successful embryo transfer and elevated β-hCG levels were the results of a personalized treatment plan that included a GnRH antagonist protocol, GnRHa trigger, AH, and the HOST. Although encouraging, the study's limitations stem from its singular case focus, highlighting the necessity for additional investigation to extrapolate these conclusions. However, these findings highlight the importance of customized approaches and advanced reproductive technologies in tackling difficult infertility issues.
